# Impact of the COVID-19 Pandemic on HPV Vaccination in Low- and Middle-Income Countries: A Scoping Review

**DOI:** 10.3390/vaccines14050432

**Published:** 2026-05-12

**Authors:** Joyce Omondi, Robert Ambogo, Candy Ochieng, Marwa Farag, George Mutwiri

**Affiliations:** 1 School of Public Health, University of Saskatchewan, Saskatoon, SK S7N 5E5, Canada; joyce.omondi@usask.ca (J.O.); qtv407@mail.usask.ca (R.A.); candy.ochieng@usask.ca (C.O.); marwa.farag@usask.ca (M.F.); 2 School of Public Administration and Development Economics, Doha Institute for Graduate Studies, Doha P.O. Box 200592, Qatar

**Keywords:** vaccination disruptions, COVID-19, programming adaptations, catch-up vaccination, vaccine delivery models, human papillomavirus, adolescent vaccination

## Abstract

**Background:** The COVID-19 pandemic caused disruptions in HPV vaccination and may have severely undermined global cervical cancer prevention, posing long-term risks to controlling cervical cancer and other HPV-related diseases. **Objective:** We conducted a scoping review to map and synthesize available evidence on how the COVID-19 pandemic has affected human papillomavirus (HPV) vaccination programs in low- and middle-income countries (LMICs) focusing on changes in vaccine delivery and coverage, determinants of uptake, economic and programmatic consequences and vaccine hesitancy. **Methods:** Inclusion criteria were limited to studies published in the English language between January 2020 to May 2025, and followed JBI and Arksey & O’Malley’s scoping review guidelines. The review proceeded through three stages: database searches, gray literature and citation tracking and used a PRISMA-ScR checklist to guide narrative and tabular synthesis. **Results:** A total of 1063 records, 57 studies were included in the final analysis, and these were spread out across 37 low- and middle-income countries (LMICs) mainly in Africa, Asia, and Latin America. Our analysis revealed that HPV vaccination coverage declined substantially during the COVID-19 pandemic, with reductions of up to 90% reported across the included studies, in the context of school closures, workforce redeployment, and supply-chain disruptions. Recovery efforts also faced major barriers including vaccine hesitancy, misinformation about COVID-19 vaccines, and travel restrictions. Strategies like digital tools, mobile clinics, and community health workers showed promise alongside integrated school- and facility-based approaches, although there is limited evidence on cost-effectiveness and long-term sustainability of these strategies. **Conclusions:** HPV vaccination in LMICs was significantly disrupted by the COVID-19 pandemic due to unreliable vaccine supply chains, health-worker shortages, and challenges tied to school-based vaccine delivery. Although recovery methods show potential, longer observation periods are needed to determine their full effectiveness.

## 1. Introduction

Cervical cancer represents the second most common cause of cancer-related deaths globally, especially within low- and middle-income countries (LMICs) and the primary causative agent of this form of cancer is the human papillomavirus (HPV); which is classified as a non-enveloped DNA virus belonging to the Papillomaviridae family [1]. Infection with HPV results in cervical cancer and several other forms of malignancy; however, it has been demonstrated that vaccination against HPV infection will prevent these forms of disease and decrease rates of HPV infection by 91% [2]. The target population for HPV vaccine administration includes girls aged 9–14, although many countries extend their eligibility to boys as well as to a catch-up group consisting of individuals up to twenty-six years of age [3]. While the efficacy of HPV vaccines in decreasing HPV infection and incidence of pre-cancerous lesions within vaccinated populations has been demonstrated, there continues to be an inconsistent level of global coverage of HPV vaccination [4,5]. Recent clinical trial and systematic review data demonstrate that a single dose of HPV vaccine provides similar levels of protection as multi-dose schedules, supporting WHO recommendations for one-or-two dose schedules and offering potential benefits for programs operating in LMICs [6]. High-income countries (HICs) have higher coverage (65%) than low- and middle-income countries (LMICs) (23.4%), even though LMICs bear approximately 80% of the global cervical cancer burden [3,4,5,7]. Prior to the COVID-19 pandemic, LMICs struggled with logistical constraints, inequitable access to healthcare services, and vaccine hesitancy [8,9,10]. Barriers to HPV vaccination in LMICs include low parental awareness, cultural myths, misconceptions, and gender-influenced decision-making structures [11].

The prolonged disruptions to health systems and vaccination programs caused by the COVID-19 pandemic led to substantial interruption in routine vaccination services [12]. According to the WHO Regional Office for Africa (WHO AFRO), childhood vaccination rates, which have already declined over the past three decades, fell further in 2021 due to pandemic-related disruptions [13]. Contributing factors included health containment measures, service delivery restrictions, supply delays, reallocation of healthcare resources, as well as misinformation and vaccine hesitancy [14,15,16,17,18]. The World Health Organization (WHO) and the United Nations International Children’s Emergency Fund (UNICEF) similarly reported that more than 25% of the HPV vaccine coverage achieved by 2019 was lost during the COVID-19 pandemic, primarily due to service disruptions, supply chain interruptions, and shifting health system priorities [13]. Globally, most regions experienced declines in coverage during 2020–2021, with a few exceptions such as Pakistan [19,20]. A recent global analysis of WHO/UNICEF HPV coverage estimates further showed that mean HPV1 program coverage among girls in low- and middle-income countries declined from about 65% in 2010–2019 to 50% in 2020–2021, while coverage in high-income countries increased over the same period, widening existing inequities [21].

Global supply chain constraints during the pandemic also adversely affected HPV vaccine availability, particularly in countries reliant on Global Alliance for Vaccines and Immunization (GAVI) supported programs [22]. COVID-19 pandemic intensified behavioral and social barriers to HPV vaccine uptake, such as distrust in health authorities and vaccine hesitancy [23,24]. Distrust in health, parental attitudes, cultural values, and fear of side effects became even more pronounced during the pandemic, especially among adolescents and their caregivers [25].

The pandemic also threatens the long-term and cost-effectiveness of HPV immunization investments by increasing missed doses and future treatment burdens [26]. Importantly, it exposed critical vulnerabilities in national adolescent immunization platforms and has prompted calls for a strategic reset in HPV vaccination delivery, advocating for stronger data systems, diversified delivery models, improved community engagement, and better integration with broader adolescent health services [27,28]. In some countries, HPV vaccination has been used as a sentinel platform to measure health system resilience and guide recovery strategies. Although the COVID-19 pandemic disrupted HPV immunization programs and resulted in missed vaccination opportunities, it also led to the adoption of innovative service delivery models, such as telehealth and hybrid outreach strategies [29]. Recovery strategies have included the use of COVID-19 vaccination hubs for HPV catch-up, mobile outreach teams, health campaigns after school reopening, and the integration of reminder systems and digital communication tools [30].

There is increasing interest in a potential association between HPV and prostate cancer, which adds urgency to reconsidering sex-neutral vaccination policies and expanding HPV prevention beyond cervical cancer [31]. This evolving evidence base may influence national program priorities and recovery plans in the post-pandemic era. Several studies have examined the impact of COVID-19 on HPV vaccination programs and have highlighted a range of effects across regions and population groups [17,20,22,28,31]. These include changes in vaccination rates, programmatic adjustments, gender-based difference in uptake, implementation of catch-up strategies, and various policy responses [22,28]. However, the literature remains fragmented, encompassing qualitative studies, program evaluations, observational data, economic models, and policy commentaries. To date, no scoping review has systematically synthesized this evidence or examined the multi-dimensional impacts of the pandemic on HPV vaccination in LMICs. Therefore, this scoping review aims to identify and synthesize the extent and nature of existing evidence on the impact of the COVID-19 pandemic on HPV vaccination programs in low- and middle-income countries (LMICs), specifically, we map: (1) reported changes in HPV vaccination coverage; (2) disruptions and adaptations to delivery platforms (including school closures and community-based strategies); (3) determinants of vaccine uptake; (4) economic and programmatic implications; and (5) strategies implemented to support program recovery.

## 2. Materials and Methods

### 2.1. Study Design and Protocol

This study followed A JBI scoping review methodology and is reported using the PRISMA-ScR Checklist [32,33,34]. The protocol was registered on the Open Science Framework (OSF).

### 2.2. Search Strategy

Multiple databases were searched, including Web of Science, Medline, Embase, Cochrane, ProQuest and Scopus databases, for articles published from January 2020 to May 2025 to gather information for this scoping review. The reviewers familiarized themselves with the data using Covidence to screen and evaluate the articles, which enhanced their understanding of recurring themes and trends. A three-step comprehensive search approach was used to identify published literature relevant to the study objectives. The database search was conducted on 12 June 2025, found in Appendix A.

(i) The preliminary search included MEDLINE (via PubMed) and Embase databases to identify relevant papers. The research team extracted keywords, index terms, and Medical Subject Headings (MeSH) to develop a comprehensive search strategy.
(ii) The finalized search strategy, guided by the research question, was then applied to additional databases, including African Journals Online (AJOL), Allied and Complementary Medicine, Cochrane Library, Index Medicus for the Eastern Mediterranean Region (IMEMR), International Bibliography of Social Sciences, LILACS, and Web of Science.
(iii) Included articles were screened for additional evidence through forward and backward citation tracking. Gray literature was accessed through Google Scholar, Web of Science conference proceedings, Open Access Theses and Dissertations (OATD), ProQuest Dissertations and Theses Global, PsycINFO and health organization repositories such as Canadian Institutes of Health Research (CIHR), National Institutes of Health (NIH), UNICEF and World Health Organization (WHO).

### 2.3. Eligibility Criteria

The eligibility criteria were based on the Population–Concept–Context (PCC) framework, Table 1.

**Table 1:** Population–Concept–Context (PCC) Framework guiding the scoping review eligibility criteria.

Component	Description
Population	People in low- and middle-income countries (LMICs) who are vaccine-eligible for HPV vaccination primarily adolescent girls aged 9–14 years, and boys in sex-neutral programs, along with catch-up age groups as defined by each jurisdiction. The population also included out-of-school adolescents, their caregivers, and key influencers such as healthcare workers, teachers, and community leaders involved in HPV vaccine uptake. Specific risk groups, such as migrants, were also included.
Concept	The focus of the study was on how COVID-19 interrupted HPV vaccination programs, examining service delivery and supply chain disruptions. In addition, the impact of COVID-19 on vaccine coverage, delivery methods, and the overall program implementation process was evaluated. The study also explored behavioral and social barriers, facilitators and recovery methods including catch-up programs, task-shifting, and service integration. Finally, it assessed the evidence supporting sustainable approaches.
Context	The research focused on LMICs, as classified by World Bank standards during the COVID-19 pandemic between January 2020 and May 2025 across various healthcare delivery settings including urban, peri-urban, rural, fragile and humanitarian environments.

### 2.4. Sources of Evidence

The diverse evidence base required was achieved through inclusion of primary studies, systematic reviews, meta-analyses, letters to the editor, policy documents and guidelines, conference proceedings, and additional gray literature sources.

### 2.5. Study Selection

The web-based Covidence platform supported systematic review management throughout the documentation process by removing duplicates and facilitating collaborative screening. Title and full-text screening were conducted to assess studies against predefined inclusion criteria. Two independent reviewers carried out the screening, and a third reviewer resolved any conflicts. All reasons for full-text exclusions were documented. The study selection process was documented using a PRISMA flow diagram shown below.

### 2.6. Data Extraction

Two reviewers developed and tested a standardized data extraction sheet. The sheet included the author’s name, year of publication, study objectives, population characteristics, sample size, concept/intervention/phenomenon of interest, outcomes, context or geographic location, and study duration. One reviewer extracted the data, and a second reviewer verified its accuracy. The extraction process led to several revisions of the framework, which are made throughout data extraction.

### 2.7. Data Analysis and Presentation

The research questions guided the use of qualitative content analysis to synthesize study findings. Results were summarized through narrative synthesis and graphical representations. This reporting system follows the PRISMA-ScR checklist to ensure transparency and comprehensive reporting, and the findings will be published in a peer-reviewed open-access journal.

## 3. Results

**Figure 1:**
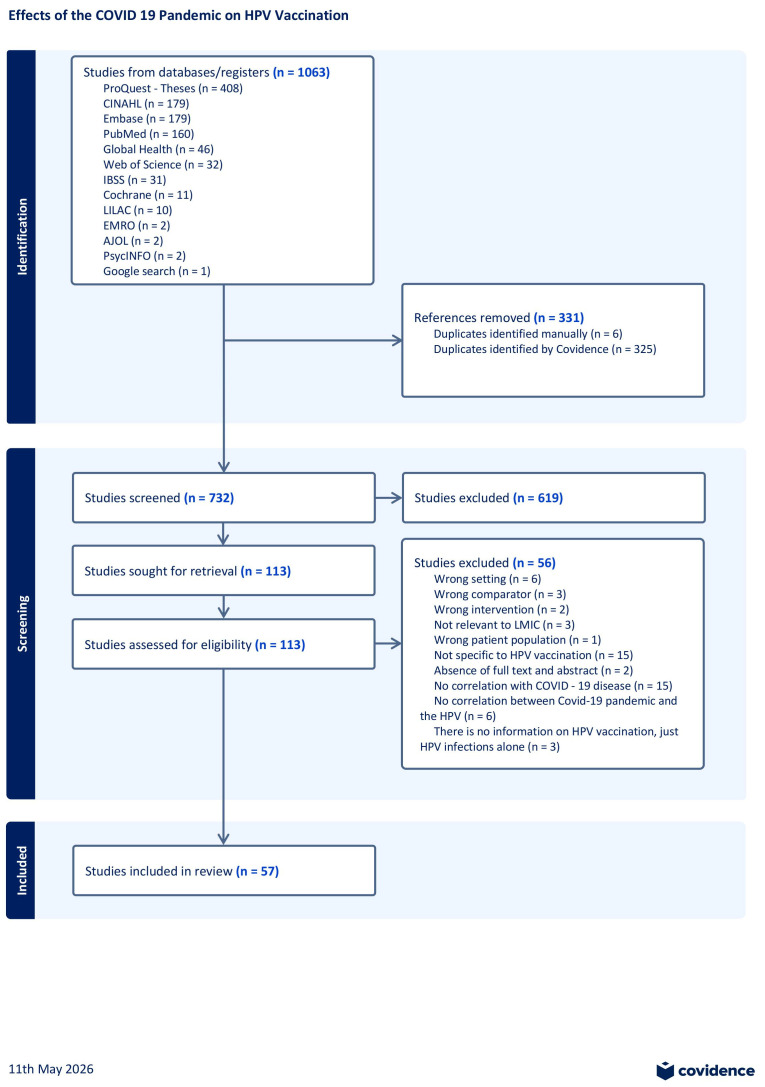
PRISM-ScR flow diagram for article selection. Of 1063 records identified, 113 full-text articles were assessed, and 57 studies were included.

A total of 1063 records were identified through database searches and gray literature sources. After duplicate removal and title and abstract screening, 113 full-text articles were assessed for eligibility, resulting in the inclusion of 57 studies in the final analysis. These comprised peer-reviewed journal articles (n = 41), professional journals (n = 10), conference reports (n = 4), institutional reports (n = 1), and national immunization updates (n = 1). The study selection process is presented in the PRISMA flow diagram (see Figure 1 in the Section 3).

### 3.1. Publication Trends

**Figure 2:**
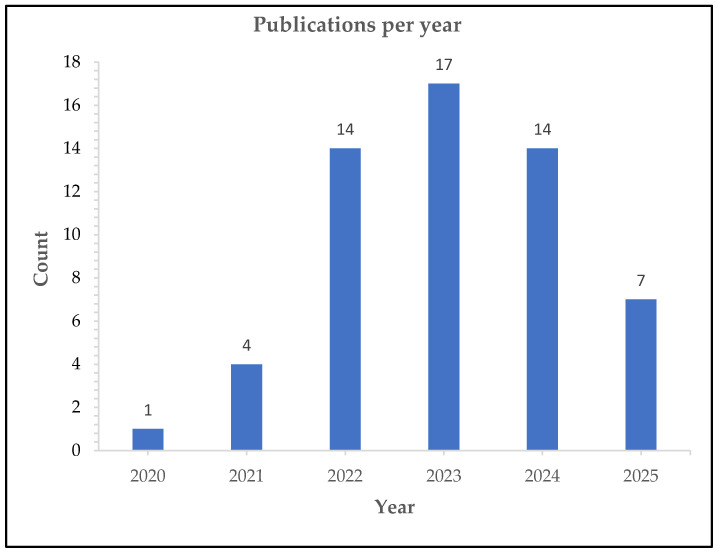
Number of included studies by year (2020–2025). Publications increased over time, peaking in 2023 (n = 17). Total n = 57 reflecting growing interest in HPV vaccination and COVID-19 in LMICs.

The included studies were published between January 2020 and May 2025, with the highest number of publications occurring in 2023 (n = 17), as seen in Figure 2.

The 57 studies were conducted across 37 LMICs, with representation from sub-Saharan Africa (n = 22), South and Southeast Asia (n = 3), and Latin America and the Caribbean (n = 9). Several multi-country studies focused on LMICs were also included (n = 20). The included research designs, including systematic and scoping reviews (n = 19), cross-sectional surveys (n = 3), qualitative evaluations (n = 19), quantitative evaluations (n = 6), and mixed-methods studies (n = 6). None of the studies reported on policy briefs (n = 0).

The target populations represented in these studies included vaccine-eligible adolescent girls aged 9–14 years and boys in sex neutral programs, their caregivers, healthcare workers, teachers, healthcare stakeholders, and clinic managers. One study focused specifically on migrants living in Ukraine (n = 1). Of the 38 studies that reported on vaccination program settings, the majority were school-based (n = 16), followed by facility-based programs (n = 6), community/outreach programs (n = 1), and mixed setting approaches (n = 3).

Across quantitative studies, most authors relied on descriptive statistics of routine immunization data or cross-sectional survey estimates, with only few employing more advanced statistical analyses or formal impact evaluations. Qualitative and mixed-method studies typically used thematic or content analysis to derive their conclusions about barriers, facilitators, and program adaptations.

**Figure 3:**
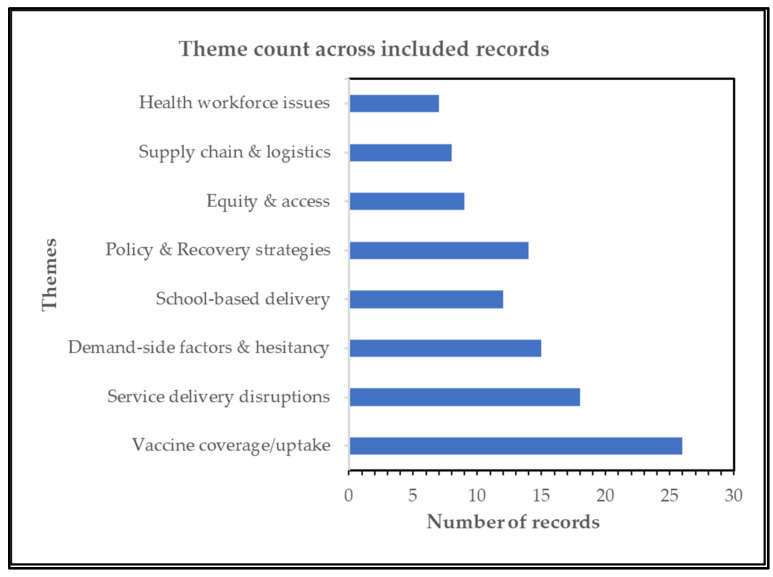
Frequency of key themes across included studies (n = 57), including inequalities, missed cohorts, recovery and catch-up strategies. Values represent the number of studies per theme; studies may contribute to multiple themes, illustrating which aspects of HPV vaccination disruption and recovery have been frequently documented in the literature.

The main themes identified across the included studies were vaccine hesitancy and demand-side issues (52.6%), interruptions in school-based vaccination delivery (29.8%), declines in vaccine coverage (26.3%), and disruptions in service delivery (35.1%). However, it is worth noting that most of the studies reported the implementation of recovery plans and policies (71.9%). The themes identified across the studies are summarized in Figure 3 below.

### 3.2. Key Findings

#### 3.2.1. Disruptions to HPV Vaccination Programs Across Regions and Populations

The COVID-19 pandemic significantly disrupted HPV vaccination initiatives (n = 38). The extent and nature of these disruptions varied by target population, service delivery approach, and geographic context.

(a) Closure of schools

School closures, reported in 45% of the included studies, had a particularly strong impact on HPV vaccination programs, especially in countries where vaccines were primarily administered in school settings. Schools were also central venues for awareness-raising and HPV vaccine promotion campaigns. A total of 12 countries in sub-Saharan Africa completely stopped school-based immunization programs for more than a year. Due to prolonged school closures, HPV vaccination was fully suspended in Zimbabwe, Kenya, and Uganda in 2020 [35]. In addition, travel restrictions further hindered vaccine delivery to rural schools, exacerbating inequities in access [36].

(b) Health workforce redeployment

Health workers responsible for immunization were reassigned to roles such as contact tracing and COVID-19 vaccination efforts [37]. Because COVID-19 response activities were prioritized, countries such as Vietnam and Peru reported reduced staffing capacity for routine vaccinations [38]. In many sub-Saharan African countries, including Malawi and Zambia, understaffing led to the temporary closure of rural health clinics [39].

(c) Supply chain interruptions

Travel restrictions, complete lockdowns, international transportation limitations, and inadequate cold-chain maintenance resources caused delays and reductions in vaccine supply to various countries and health facilities, leading to interruptions in vaccination activities. In countries such as Pakistan, Bangladesh and Nepal, where non-COVID vaccine storage and transportation were de-prioritized, significant vaccine stock depletion was reported [40].

(d) Differential impact by population group

A study conducted in Ukraine among immigrants affected by the Ukraine–Russia conflict found that adolescents who were not enrolled in formal education, as well as girls over the age of 15, were disproportionately excluded from catch-up vaccination efforts particularly in informal settlements and refugee camps. As a result, many adolescent girls did not receive HPV vaccination during the COVID pandemic. In Zambia, patriarchal family structures meant that health-seeking decisions such as vaccinations of daughters were de-prioritized during periods of economic hardship, restricting adolescent girls’ access to HPV vaccination due to gendered care dynamics [41].

#### 3.2.2. Impact on HPV Vaccine Coverage, Service Models, and Program Implementation

(a) Decline in vaccine coverage

Sharp declines in immunization coverage were documented in many of the included studies (86.4%). Mohamed et al. (2025) reported an almost 15% decrease across Latin America [42]. In Kenya, first-dose uptake decreased by 65% in 2020 compared to 2019 [43]. National reporting systems indicated that HPV vaccine distribution in India in 2020 was 50% lower than in 2019 [44]. Although partial recovery was observed by late 2022, often driven by small-scale catch-up initiatives, full restoration remained challenging.

(b) Collapse of service delivery models

School-based immunizations and a multi-platform delivery approach combining health-facility- and school-based vaccination strategies were the two primary delivery mechanisms identified in the review. The most vulnerable model was the school-based approach, which predominated in many LMICs, including Kenya, Liberia and Zambia where nationwide school closures halted routine vaccination [10]. Countries employing multi-platform delivery, such as Rwanda, demonstrated greater resilience and faster recovery [45]. In contrast, countries that relied exclusively on school-based vaccination faced challenges in adapting due to prolonged closures and heightened vaccine hesitancy [46].

(c) Change in resource prioritization

Included studies also reported that governments prioritized COVID-19 response funding over HPV interventions, particularly in LMICs such as Kenya, Zambia, Cote d’Ivoire and Malawi [47]. As a result, HPV vaccination was labeled non-essential, leading to reduced budgets and staffing constraints [48].

#### 3.2.3. Barriers and Facilitators to HPV Vaccine Uptake

(a) Barriers

Of the 57 included studies, 23 reported barriers that limited HPV vaccination uptake. Key barriers included:

1. Behavioral and social factors
  - The fear of contracting COVID-19 reduced public willingness to visit medical institutions [35].
  - Misconceptions and misinformation: widespread misinformation about HPV and COVID-19 vaccination circulated across Latin America, and Sub-Saharan Africa, including Kenya and Cameroon [35].
2. Infrastructure, logistical and economic factors
  - Four studies reported that lockdown-related transport restrictions hindered access to medical facilities and disrupted vaccination distribution.
  - Three studies indicated that LMICs governments faced financial difficulties, leading to reduced motivation among healthcare staff.
  - During the COVID-19 pandemic, adolescent schoolgirls missed HPV vaccines due to school closures, as documented in most studies (n = 31).
3. Cultural factors

Seven studies reported that increased parental and caregiver reluctance to encourage HPV immunizations was driven by fear of COVID-19 exposure and mistrust of public health measures during the COVID-19 pandemic [49].

(b) Facilitators

Despite the challenges, several facilitators for HPV vaccination were identified across 43 studies:

  - Community health workers: In Nepal and Zimbabwe, these workers played essential roles in building awareness and trust.
  - Policy coordination and integration with COVID-19 response: For example, Rwanda, integrated HPV vaccination days with COVID-19 awareness campaigns, leveraging existing outreach infrastructure [45].
  - Digital technologies: Tools such as telemedicine, mobile-based appointments systems [40], reminders, messaging and alternate delivery methods were tested in India and Ukraine [50]; nonetheless, their efficacy was low in rural regions [45].
  - Community outreach: Education campaigns aiming at informing the public about the significance of HPV vaccination were seen through government officials, medical professionals, and barazas. These strategies were seen in Kenya and Colombia [43,51].

#### 3.2.4. Strategies to Maintain or Restore HPV Vaccination and Their Effectiveness

Few studies (n = 7) detailed initiatives aimed at helping adolescent girls who missed HPV immunizations during the pandemic return to routine services through catch-up programs.

1. Catch-up vaccination campaigns and sensitizations

Following the reopening of schools, Rwanda and Zambia implemented catch-up initiatives in both schools and community settings [40]. By late 2023, Rwanda had restored its HPV vaccination coverage to 90% of pre-pandemic levels [52].

2. Use of alternative delivery platforms

In countries such as Kenya and Pakistan, mobile clinics and telemedicine approaches were used to reach underprivileged girls outside of formal school systems [52]. In India, Adolescent Health Days incorporated HPV vaccination and nutrition programs to expand reach [40,44].

3. Partnership with communities and non-governmental organizations

After the pandemic, NGOs played a key role in reviving HPV vaccination programs in Malawi and Kenya, focusing on community mobilization and addressing vaccine skepticism [53].

4. Evaluation of the recovery strategies

**Figure 4:**
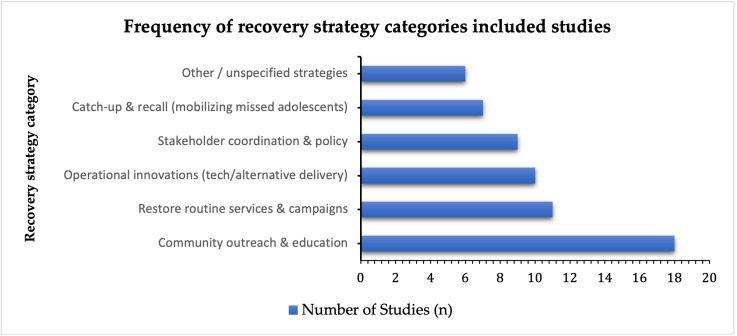
Frequency of reported HPV vaccination recovery strategies (n = 57). Values indicate the number of studies reporting each strategy; multiple strategies may be reported within a single study, highlighting which types of recovery approaches (such as catch-up campaigns or alternative platforms) have been commonly used in LMICS.

Despite these efforts, most recovery techniques lacked quantitative evaluation. Few studies provided evaluations of sustainability, cost-effectiveness, and overall impact. The most well-documented example of recovery was Rwanda, attributed to its integrated school health model, strong community trust, and centralized coordination, as seen in Figure 4.

## 4. Discussion

This scoping review synthesized evidence from 57 studies evaluating how the COVID-19 pandemic affected human papillomavirus (HPV) vaccinations for eligible populations in low–middle-income countries (LMICs). The findings indicate there has been an unprecedented, widespread and multifaceted disruption to HPV vaccine delivery programs due to COVID-19-related factors including school closure, increased burden on the health system and redeployment of healthcare workers, and behavioral reluctance due to fear or misinformation surrounding the pandemic.

Across the 37 articles focused on LMICs, HPV vaccination rates declined significantly from 80% to 20% during the early pandemic period (mainly 2020–2021) [35], and particularly in those countries that utilized school-based delivery strategies [54]. The extent of disruption to HPV vaccination programs varied depending on countries geographical location, level of development in their health infrastructure, and flexibility of existing HPV vaccine delivery platforms. The COVID-19 pandemic also highlighted inherent vulnerabilities in HPV vaccination program design, particularly in developing countries with very little community engagement mechanisms or flexible delivery platforms to address such disruptions (e.g., Kenya) [46]. Despite the many challenges posed by the pandemic, several countries such as Brazil, Ukraine and Rwanda have employed mitigation measures such as: sending digital reminders regarding appointments, integrating HPV vaccines into ongoing COVID-19 response efforts, and utilizing mobile outreach clinics [36,50]. While the majority of the literature describing these types of strategies are descriptive based on routine coverage data or small observational studies, very few studies evaluated the impacts of these interventions through randomized controlled trials or comparative analysis. This limits the ability to draw strong conclusions about the relative effectiveness and generalizability of different methods to recover from these disruptions.

Overall, due to the dominance of the evidence base being composed of observational study designs, routine programmatic data, and qualitative accounts, the synthesis described here can identify consistent patterns and outline areas of need; however, we are unable to assess causality associated with specific disruptions or recovery strategies.

### 4.1. School-Based Delivery Models

One important finding from this review is the heavy reliance on school-based delivery models for HPV vaccination programs in LMICs, as shown in similar studies [54]. While these models worked well in normal circumstances, they were extremely susceptible when schools were closed, especially in South Asia and sub-Saharan Africa [52]. As a result, HPV vaccination rates in countries such as Kenya and India nearly collapsed during the first year of the pandemic [43,44]. This over-dependence on a single delivery mechanism left little room for rapid adaptation. Greater resilience was higher in countries with more diversified or integrated delivery methods (e.g., integrating outreach, facility-based, and school-based programs) [55]. Unlike Uganda and Sierra Leone, where catch-up efforts took longer [35], Rwanda recovered vaccination coverage more rapidly, aided by using community health workers and school-health programs [55].

### 4.2. Health System Trade-Offs

Health systems had to rearrange their priorities during the pandemic, frequently neglecting preventative treatments like HPV vaccination [56]. Routine vaccination programs had trade-offs because of the reallocation of cold chain resources [55], the redeployment of immunization staff, and underbudgeting to channel funds toward the COVID-19 response [56]. These compromises highlight a recurring issue in LMICs: insufficient buffering capacity of the health system. During crises, essential services are less prioritized in the absence of surge support or backup preparations. The WHO’s global objectives to eliminate cervical cancer, depends heavily on improving HPV vaccination rates in LMICs, [57].

### 4.3. Behavioral, Cultural and Social Factors

During the pandemic, behavioral and social factors had a significant impact on HPV vaccination uptake [58], despite logistical constraints being the major setback [49]. In other countries, vaccine uptake was disrupted by misinformation, increased caregiver hesitation, and fear of infection [59]. Notably, misinformation was reported in Southeast Asia, West and East Africa that connected HPV vaccinations with COVID-19 contamination [60]. In certain patriarchal communities [59], families prioritized their essential needs over the health of adolescent girls [51]. This implies that, in addition to technical delivery capabilities, attempts to improve HPV vaccination in LMICs should take sociocultural considerations into account [61].

### 4.4. Mitigation Strategies

Certain nations showed creativity and adaptability in maintaining HPV immunization [62]. The strategies that were most often mentioned were integration with COVID-related services, mobile vaccination units, and digital outreach platforms [40]. Nevertheless, comprehensive reviews and cost-effectiveness studies of these strategies were lacking [63]. Furthermore, many mitigation strategies concentrated on short-term recovery rather than addressing underlying structural weaknesses, such as inadequate adolescent-focused health infrastructure, insufficient vaccination monitoring systems, and a shortage of human resources [64].

While the COVID-19 pandemic resulted in significant reductions in HPV vaccine coverage across a wide range of low–middle-income countries (LMICs), there are reports from multiple studies suggesting that recovery occurred after the easing of restrictions when schools and community services reopened [13,21]. For example, while Rwanda was able to use both catch-up campaigns and school/community outreach as part of their overall school health program to bring coverage back up to near levels seen before the pandemic, other countries were unable to achieve full recovery and continue to have significantly lower than baseline coverage rates [13,52]. Overall, it appears that most of the evidence is suggestive of varying recovery patterns based on the specific contexts within each country, although countries that had used more diverse delivery systems had engaged with communities, and appeared to be recovering at a faster rate [13,21,52].

## 5. Implications

### 5.1. Policy Implications

Governments in LMICs must re-strategize HPV vaccination delivery strategies considering pandemic-related challenges [48]. Policy shifts should favor flexible, multi-platform delivery models that combine both school-based, health facilities, and community-based mechanisms, to ensure continuity during public health crises. There is also a need for policy alignment between HPV and broader adolescent health programs, integrating services like nutrition, menstrual hygiene, and health education to improve uptake and efficiency [62,65].

### 5.2. Practice Implications

Healthcare systems should prioritize:

(a) Training of community health workers to conduct HPV vaccine outreach.
(b) Expanding eligibility tracking systems beyond school registers.
(c) Strengthening communication strategies to combat vaccine misinformation and rebuild public trust.
(d) Inclusion of adolescent-focused health services in emergency preparedness planning is essential.
(e) Routine immunization programs must be protected during crisis response efforts to avoid long-term setbacks.

### 5.3. Research Gaps

This review showed that little is known about the cost, sustainability, and equity of catch-up campaigns used during and after the COVID-19 pandemic for the HPV vaccinations [12]. Therefore, the study recommends future studies to be done to evaluate the effectiveness of recovery strategies used during and after the COVID-19 pandemic period. In addition, given the increasing interest in one-dose HPV vaccination schedule, and recent WHO recommendations allowing one-or-two dose regimens for many age groups, further research in studies are needed to assess their real-world effectiveness and programmatic performance in the context of low- and middle-income countries (LMICs).

## 6. Strengths and Limitations of the Study

### 6.1. Strengths

The study has conducted a comprehensive synthesis of global evidence on a critical yet underexplored topic. In addition, the use of a scoping review methodology allowed inclusion of diverse study designs, including gray literature and programmatic reports.

### 6.2. Limitations

The diversity of study designs, data sources and outcome measures made it difficult to compare results directly or to quantitatively synthesize effect sizes across studies. Furthermore, because many included studies were descriptive and did not perform formal impact evaluations or detailed statistical analyses, the robustness of reported effects and conclusions is variable and should be interpreted with caution.

## 7. Conclusions

HPV vaccination efforts in LMICs were severely disrupted by the COVID-19 pandemic, exposing weaknesses in vaccine supply chains and the dependence on school-based delivery programs. HPV vaccination was worsened by social and behavioral factors, such as vaccine hesitancy, fear of infection, and vaccine misinformation. There is a need for more adaptable, context-sensitive methods that go beyond schools and give priority to adolescent health as LMICs recover and adjust. Elimination of cervical cancer will require the presence of inclusive, planned recovery measures for HPV vaccination post-pandemic.

## Data Availability

Data sharing not applicable.

## Abbreviations

The following abbreviations are used in this manuscript:

LMICs	Low- and middle-income countries
HPV	Human Papillomavirus
HICs	High Income Countries
WHO	World Health Organization
UNICEF	United Nations International Children’s Emergency Fund
GAVI	Global Alliance for Vaccines and Immunization

## Appendix A

**Concept 1: HPV Vaccination**	**Concept 2: COVID-19 Pandemic**	**Concept 4: Vaccine Delivery**
“HPV vaccine”	“COVID-19”	“Vaccine delivery”
“HPV vaccine *”	“Coronavirus disease 2019”	“Immunization delivery”
“Human papillomavirus vaccine”	“SARS-CoV-2”	“Vaccination program *”
“HPV immunization”	“COVID-19 pandemic”	“Healthcare delivery”
“Papillomavirus vaccine”	“Novel coronavirus”	“Service delivery”
“Cervarix”	“Pandemic response”	“Routine immunization”
“Gardasil”		“School-based vaccination”
* Search concepts used in the ProQuest Theses, CINAHL, Embase, PubMed, Global Health, Web of Science, IBSS, Cochrane, LILAC, EMRO, AJOL, PsycINFO, and Google search databases.		

## References

[1] Rodrigues C.M.C., Plotkin S.A. (2020). Impact of Vaccines; Health, Economic and Social Perspectives. Front. Microbiol..

[2] Plotkin S.A. (2005). Vaccines: Past, present and future. Nat. Med..

[3] Cheng L., Wang Y., Du J. (2020). Human Papillomavirus Vaccines: An Updated Review. Vaccines.

[4] De Martel C., Plummer M., Vignat J., Franceschi S. (2017). Worldwide burden of cancer attributable to HPV by site, country and HPV type. Int. J. Cancer.

[5] Bruni L., Diaz M., Barrionuevo-Rosas L., Herrero R., Bray F., Bosch F.X., de Sanjosé S., Castellsagué X. (2016). Global estimates of human papillomavirus vaccination coverage by region and income level: A pooled analysis. Lancet Glob. Health.

[6] Whitworth H.S., Mounier-Jack S., Choi E.M., Gallagher K.E., Howard N., Kelly H., Mbwanji G., Kreimer A.R., Basu P., Barnabas R. (2024). Efficacy and immunogenicity of a single dose of human papillomavirus vaccine compared to multidose vaccination regimens or no vaccination: An updated systematic review of evidence from clinical trials. Vaccine X.

[7] Sabeena S., Bhat P.V., Kamath V., Arunkumar G. (2018). Global human papilloma virus vaccine implementation: An update. J. Obstet. Gynaecol. Res..

[8] MacDonald N.E., Eskola J., Liang X., Chaudhuri M., Dube E., Gellin B., Goldstein S., Larson H., Manzo M.L., Reingold A. (2015). Vaccine Hesitancy: Definition, Scope and Determinants. Vaccine.

[9] Zheng L., Wu J., Zheng M. (2021). Barriers to and Facilitators of Human Papillomavirus Vaccination Among People Aged 9 to 26 Years: A Systematic Review. Sex. Transm. Dis..

[10] Kutz J.-M., Rausche P., Gheit T., Puradiredja D.I., Fusco D. (2023). Barriers and facilitators of HPV vaccination in sub-saharan Africa: A systematic review. BMC Public Health.

[11] Petersen Z., Jaca A., Ginindza T.G., Maseko G., Takatshana S., Ndlovu P., Zondi N., Zungu N., Varghese C., Hunting G. (2022). Barriers to uptake of cervical cancer screening services in low-and-middle-income countries: A systematic review. BMC Women’s Health.

[12] Toh Z.Q., Kosasih J., Russell F.M., Garland S.M., Mulholland E.K., Licciardi P.V. (2019). Recombinant human papillomavirus nonavalent vaccine in the prevention of cancers caused by human papillomavirus. Infect. Drug Resist..

[13] WHO (2022). COVID-19 Pandemic Fuels Largest Continued Backslide in Vaccinations in Three Decades.

[14] Torner N. (2023). The end of COVID-19 public health emergency of international concern (PHEIC): And now what?. Vacunas.

[15] Eurosurveillance Editorial Team (2020). Note from the editors: World Health Organization declares novel coronavirus (2019-nCoV) sixth public health emergency of international concern. Eurosurveillance.

[16] Vraga E.K., Brady S.S., Gansen C., Khan E.M., Bennis S.L., Nones M., Tang R., Srivastava J., Kulasingam S. (2023). A review of HPV and HBV vaccine hesitancy, intention, and uptake in the era of social media and COVID-19. eLife.

[17] D’Amato S., Nunnari G., Trimarchi G., Squeri A., Cancellieri A., Squeri R., Pellicanò G.F. (2022). Impact of the COVID-19 pandemic on HPV vaccination coverage in the general population and in PLWHs. Eur. Rev. Med. Pharmacol. Sci..

[18] Zimmerman T., Shiroma K., Fleischmann K.R., Xie B., Jia C., Verma N., Lee M.K. (2023). Misinformation and COVID-19 vaccine hesitancy. Vaccine.

[19] Rahman S.U., Haq F.U., Imran M., Shah A., Bibi N., Khurshid R., Romman M., Gaffar F., Khan M.I. (2021). Impact of the COVID-19 lockdown on routine vaccination in Pakistan: A hospital-based study. Hum. Vaccines Immunother..

[20] Mennini F.S., Silenzi A., Marcellusi A., Conversano M., Siddu A., Rezza G. (2022). HPV Vaccination during the COVID-19 Pandemic in Italy: Opportunity Loss or Incremental Cost. Vaccines.

[21] Casey R.M., Akaba H., Hyde T.B., Bloem P. (2024). COVID-19 pandemic and equity of global human papillomavirus vaccination: Descriptive study of World Health Organization-Unicef vaccination coverage estimates. BMJ Med..

[22] Shet A., Carr K., Danovaro-Holliday M.C., Sodha S.V., Prosperi C., Wunderlich J., Wonodi C., Reynolds H.W., Mirza I., Gacic-Dobo M. (2021). Impact of the SARS-CoV-2 pandemic on routine immunisation services: Evidence of disruption and recovery from 170 countries and territories. Lancet Glob. Health.

[23] Aguolu O.G., Malik A.A., Ahmed N., Omer S.B. (2022). Overcoming Vaccine Hesitancy for Future COVID-19 and HIV Vaccines: Lessons from Measles and HPV Vaccines. Curr. HIV/AIDS Rep..

[24] Lazarus J.V., Wyka K., White T.M., Picchio C.A., Rabin K., Ratzan S.C., Leigh J.P., Hu J., El-Mohandes A. (2022). Revisiting COVID-19 vaccine hesitancy around the world using data from 23 countries in 2021. Nat. Commun..

[25] Holman D.M., Benard V., Roland K.B., Watson M., Liddon N., Stokley S. (2014). Barriers to human papillomavirus vaccination among US adolescents: A systematic review of the literature. JAMA Pediatr..

[26] Troiano G., Nardi A. (2021). Vaccine hesitancy in the era of COVID-19. Public Health.

[27] Gilkey M.B., Bednarczyk R.A., Gerend M.A., Kornides M.L., Perkins R.B., Saslow D., Sienko J., Zimet G.D., Brewer N.T. (2020). Getting Human Papillomavirus Vaccination Back on Track: Protecting Our National Investment in Human Papillomavirus Vaccination in the COVID-19 Era. J. Adolesc. Health.

[28] Toh Z.Q., Russell F.M., Garland S.M., Mulholland E.K., Patton G., Licciardi P.V. (2021). Human papillomavirus vaccination after COVID-19. JNCI Cancer Spectr..

[29] Musumeci A.E. (2022). Increasing Human Papillomavirus Vaccination Intent and Knowledge Through an Educational Intervention. Doctoral Dissertation.

[30] NCIR (2023). COVID Impact on Schools Final Report April 2023.

[31] Morka N., Norris J.M., Emberton M., Kelly D. (2021). Prostate cancer and the human papilloma virus: Causative association, role of vaccines, and the impact of the COVID-19 pandemic. Prostate Cancer Prostatic Dis..

[32] Peters M.D.J., Godfrey C., Mcinerney P., Munn Z., Trico A.C., Khalil H. (2020). Scoping Reviews. JBI Manual for Evidence Synthesis.

[33] Tricco A.C., Lillie E., Zarin W., O’Brien K.K., Colquhoun H., Levac D., Moher D., Peters M.D.J., Horsley T., Weeks L. (2018). PRISMA Extension for Scoping Reviews (PRISMA-ScR): Checklist and Explanation. Ann. Intern. Med..

[34] McGowan J., Sampson M., Salzwedel D.M., Cogo E., Foerster V., Lefebvre C. (2016). PRESS Peer Review of Electronic Search Strategies: 2015 Guideline Statement. J. Clin. Epidemiol..

[35] Merrell K., Ochieng P., Osei-Bonsu E.B., Seife E., Kemper K., Begna K., Bussman S., Leavitt T., Acheamfour O., Vanderpuye V. (2021). 1622P The impact of COVID-19 on cancer treatment delivery in Sub-Saharan Africa. Ann. Oncol..

[36] Da Silva T.M.R., de Sá A.C.M.G.N., Beinner M.A., Abreu M.N.S., Matozinhos F.P., Sato A.P.S., Vieira E.W.R. (2022). Impact of the COVID-19 Pandemic on Human Papillomavirus Vaccination in Brazil. Int. J. Public Health.

[37] Murewanhema G., Musuka G., Dzinamarira T. (2022). Facilitating sexual and reproductive health services for adolescent girls in the COVID-19 era: An urgent public health priority. Afr. J. Prim. Health Care Fam. Med..

[38] Parellada C., Felsher M., Valenzuela G., Flores C., Farias L., Saxena K. (2023). COVID-19 Impact on Human Papillomavirus Immunization Program in Peru: Modelling Time and Catch-Up Rates to Close the Immunization Gap. Int. J. Infect. Dis..

[39] Moucheraud C., Whitehead H.S., Songo J., Szilagyi P.G., Hoffman R.M., Kaunda-Khangamwa B.N. (2023). Malawian caregivers’ experiences with HPV vaccination for preadolescent girls: A qualitative study. Vaccine X.

[40] Lee J., Ismail-Pratt I., Machalek D.A., Kumarasamy S., Garland S.M. (2025). Correction: From barriers to opportunities from COVID-19 pandemic: Stakeholder perspectives on cervical cancer screening programs in LMICs of the Asia-Pacific region. PLoS Glob. Public Health.

[41] Lubeya M.K., Chibwesha C.J., Mwanahamuntu M., Mukosha M., Vwalika B., Kawonga M. (2023). Determinants of the Implementation of Human Papillomavirus Vaccination in Zambia: Application of the Consolidated Framework for Implementation Research. Vaccines.

[42] Mohamed Y., Luey E., Kata ’.U., Tukia O., Lotulelei S., Tei A., ‘Ofanoa R., Overmars I., Frawley J., Vodonaivalu L. (2025). “The only vaccine that we really question is the new vaccine”: A qualitative exploration of the social and behavioural drivers of human papillomavirus (HPV) vaccination in Tonga. Vaccine.

[43] Essoh T.-A., Adeyanju G.C., Adamu A.A., Tall H., Aplogan A., Tabu C. (2023). Exploring the factors contributing to low vaccination uptake for nationally recommended routine childhood and adolescent vaccines in Kenya. BMC Public Health.

[44] Bénard É., Drolet M., Laprise J.F., Jit M., Prem K., Boily M.C., Brisson M. (2023). Potential benefit of extended dose schedules of human papillomavirus vaccination in the context of scarce resources and COVID-19 disruptions in low-income and middle-income countries: A mathematical modelling analysis. Lancet Glob. Health.

[45] Adeyanju G.C., Essoh T.-A., Sidibe A.R., Kyesi F., Aina M. (2024). Human Papillomavirus Vaccination Acceleration and Introduction in Sub-Saharan Africa: A Multi-Country Cohort Analysis. Vaccines.

[46] Kariuki V.W., Nduati R., Wangombe A. (2024). Factors Associated with Human Papillomavirus Vaccine Uptake in Adolescents Aged 10–12 Years in Kiambu Sub-County, Kenya. Iconic Res. Eng. J..

[47] Kouassi K.S., Chisupa E., Clarke A., Massenon I., Miano C., Mutuku F., Wanyoike S., Mumba M., Diouf R., Morgan C. (2024). Pandemic-related resilience in HPV vaccination programmes–Perspectives from selected countries in Africa on what it will take to vaccinate 90% of girls by 2030. Vaccine.

[48] Guillaume D., Waheed D.-E., Schleiff M., Muralidharan K.K., Vorsters A., Limaye R.J. (2024). Global perspectives of determinants influencing HPV vaccine introduction and scale-up in low- and middle-income countries. PLoS ONE.

[49] Ginsburg O., Basu P., Kapambwe S., Canfell K. (2021). Eliminating cervical cancer in the COVID-19 era. Nat. Cancer.

[50] Ganczak M., Kalinowski P., Pasek O., Duda-Duma Ł., Sobieraj E., Goławski J., Biesiada D., Jansen D., Vervoort J.P.M., Edelstein M. (2022). Health System Barriers to Child Mandatory and Optional Vaccination among Ukrainian Migrants in Poland in the Context of MMR and HPV Vaccines—A Qualitative Study. Int. J. Environ. Res. Public Health.

[51] Cordoba-Sanchez V., Lemos M., Tamayo-Lopera D.A., Gorin S.S. (2022). HPV-Vaccine Hesitancy in Colombia: A Mixed-Methods Study. Vaccines.

[52] O’BRien K.L., Lemango E. (2023). The big catch-up in immunisation coverage after the COVID-19 pandemic: Progress and challenges to achieving equitable recovery. Lancet.

[53] Borda H., Bloem P., Akaba H., Guillaume D., Willens V., Jurgensmeyer M., Muralidharan K., Limaye R. (2024). Status of HPV disease and vaccination programmes in LMICs: Introduction to special issue. Vaccine.

[54] Zou Z., Zhang L. (2023). The one-dose schedule opens the door to rapid scale-up of HPV vaccination. BMC Med..

[55] Kasonia K., Tindanbil D., Kitonsa J., Baisley K., Zalwango F., Enria L., Mansaray A., James M., Nije Y., Tata D.T. (2023). The impact of the COVID-19 pandemic on the provision & utilisation of primary health care services in Goma, Democratic Republic of the Congo, Kambia district, Sierra Leone & Masaka district, Uganda. PLoS ONE.

[56] Rao S.R., Kampan N., Chew K.T., Shafiee M.N. (2022). The impact of the COVID-19 pandemic on the national HPV immunization pro-gram in Malaysia. Front. Public Health.

[57] WHO Cervical Cancer Elimination Initiative. https://www.who.int/initiatives/cervical-cancer-elimination-initiative.

[58] Flynn P.M., Stull C., Jain V.M., Evans M.D. (2025). A national cross-sectional study of dentists’ vaccine hesitancy and intention to provide HPV vaccines following emergency COVID-19 vaccination authorization. Vaccine.

[59] Haddison E.C., Engoung D.B., Bodo C.B., Njie V.M. (2025). Overcoming HPV vaccine hesitancy: Insights from a successful school-based vaccination campaign in the Saa health district of Cameroon. BMC Infect. Dis..

[60] Unfried K., Priebe J. (2024). Vaccine hesitancy and trust in sub-Saharan Africa. Sci. Rep..

[61] Wang Z., Chen S., Fang Y. (2022). Parental Willingness and Associated Factors of Pediatric Vaccination in the Era of COVID-19 Pandemic: A Systematic Review and Meta-Analysis. Vaccines.

[62] Karanja-Chege C.M. (2022). HPV Vaccination in Kenya: The Challenges Faced and Strategies to Increase Uptake. Front. Public Health.

[63] Domgue J.F., Dille I., Kapambwe S., Yu R., Gnangnon F., Chinula L., Murenzi G., Mbatani N., Pande M., Sidibe F. (2024). HPV vaccination in Africa in the COVID-19 era: A cross-sectional survey of healthcare providers’ knowledge, training, and recommendation practices. Front. Public Health.

[64] Cruz-Valdez A., Palacio-Mejía L.S., Quezada-Sánchez A.D., Hernández-Ávila J.E., Galicia-Carmona T., Cetina-Pérez L.d.C., Arango-Bravo E.A., Isla-Ortiz D., Aranda-Flores C.E., Uscanga-Sánchez S.-R. (2023). Cervical cancer prevention program in Mexico disrupted due to COVID-19 pandemic: Challenges and opportunities. Front. Oncol..

[65] Ndiaye C., Kyesi F., Masupha T., Ranyali M., Engel D., Guillaume D., Wanyoike S., Giattas M.R., Morgan C., Jennings M.C. (2024). Integrating HPV vaccine service delivery with adolescent health programmes–Experiences and perspectives from selected countries in Africa. Vaccine.

